# Trastuzumab Deruxtecan in HER2-Positive Metastatic Breast Cancer: Real-World Outcomes and Treatment Transition Patterns in a Chinese Cohort

**DOI:** 10.3390/curroncol33080487

**Published:** 2026-08-18

**Authors:** Yifei Chen, Ruyan Zhang, Ran Ran, Yaxin Liu, Jiayang Zhang, Ying Yan, Bin Shao, Xu Liang, Hanfang Jiang, Lijun Di, Guohong Song, Huiping Li

**Affiliations:** Key Laboratory of Carcinogenesis and Translational Research (Ministry of Education/Beijing), Department of Breast Oncology, Peking University Cancer Hospital & Institute, Beijing 100142, China; chenyifeieven@126.com (Y.C.); zhangruyan2016@163.com (R.Z.);

**Keywords:** HER2-positive breast cancer, metastatic breast cancer, trastuzumab deruxtecan, real-world study, TKI pretreated, maintenance therapy

## Abstract

We retrospectively analyzed consecutive patients with HER2-positive metastatic breast cancer who received T-DXd at a single Chinese cancer center between March 2023 and March 2026. Progression-free survival (PFS), overall survival (OS), systemic and intracranial response, safety, prognostic factors, and exploratory subsequent treatment strategies were assessed. Among 173 eligible patients, 80.3% had received prior HER2-directed TKIs and 41.6% had CNS metastases. After a median follow-up of 14.3 months, median PFS and OS were 14.1 and 31.1 months, respectively. The objective response rate was 54.5% among 154 patients with measurable systemic disease, while the CNS objective response rate was 46.3% among 67 patients with measurable CNS lesions. Among patients with measurable CNS lesions, the CNS-ORR was comparable between patients with and without peri-T-DXd local treatment (53.8% vs. 44.4%, *p* = 0.21). Greater prior treatment exposure was associated with shorter PFS, whereas HER2 IHC 3+ disease was independently associated with longer OS and showed a trend toward improved PFS compared with HER2 IHC 2+/FISH-positive disease. Interstitial lung disease occurred in 13 patients (7.5%), all grade 1–2. Among 148 patients included in the maintenance analysis, 23 switched to alternative maintenance regimens after initial benefit from T-DXd. In an exploratory analysis, no statistically significant differences in PFS or OS were observed between patients who continued T-DXd and those who switched to maintenance therapy. However, these findings require cautious interpretation because of immortal-time bias, selection bias, and limited sample size. T-DXd demonstrated clinically meaningful systemic and intracranial activity with manageable toxicity in this heavily pretreated real-world cohort.

## Simple Summary

Trastuzumab deruxtecan is an important treatment for patients with HER2-positive metastatic breast cancer, but patients treated in routine practice often have more extensive prior treatment and more frequent brain metastases than those enrolled in clinical trials. In this real-world study from China, trastuzumab deruxtecan showed meaningful activity both outside and inside the brain in a heavily pretreated population, most of whom had previously received HER2-targeted tyrosine kinase inhibitors. Side effects were generally manageable. We also explored outcomes in patients who changed to alternative maintenance treatment after initially benefiting from trastuzumab deruxtecan. These findings provide practical evidence to support treatment selection, monitoring, and future studies of treatment sequencing in HER2-positive metastatic breast cancer.

## 1. Background

Human epidermal growth factor receptor 2 (HER2)-positive metastatic breast cancer remains a clinically aggressive subtype despite major advances in HER2-targeted therapy. Trastuzumab deruxtecan (T-DXd) is a HER2-directed ADC that has become a standard treatment option after demonstrating superior efficacy over trastuzumab emtansine in the DESTINY-Breast03 trial [1].

More recently, the treatment landscape has continued to evolve with the phase III DESTINY-Breast09 trial [2], which evaluated T-DXd plus pertuzumab in the first-line setting for HER2-positive advanced or metastatic breast cancer. These findings further support the strong antitumor activity of T-DXd and suggest that its optimal use may extend to earlier lines of therapy. As T-DXd moves into earlier treatment lines and produces more prolonged disease control, long-term treatment management, including dose modification, treatment discontinuation, and possible transition to maintenance therapy, is becoming increasingly relevant in routine practice.

T-DXd has also demonstrated substantial intracranial activity as reported in the TUXEDO-1 [3] and DESTINY-Breast12 studies [4], an important consideration given that CNS metastases develop in 35–50% of patients with HER2-positive metastatic breast cancer [4,5]. However, patients enrolled in prospective trials may not fully represent routine clinical populations, particularly those with extensive prior exposure to HER2-directed tyrosine kinase inhibitors (TKIs).

Real-world patients are often more heterogeneous than trial populations, with heavier pretreatment, variable performance status, frequent CNS involvement, individualized dose modifications, and financial considerations [6,7,8,9]. In China, the widespread use of HER2-directed TKIs such as pyrotinib further distinguishes routine treatment sequences from those in many international trials. In addition, little is known about outcomes in patients who initially benefit from T-DXd but subsequently switch to alternative maintenance regimens. Therefore, we conducted a single-center retrospective real-world study to evaluate the effectiveness, safety, and intracranial activity of T-DXd in patients with HER2-positive metastatic breast cancer treated in routine clinical practice, most of whom had received prior HER2-directed TKIs. We also explored clinicopathologic factors associated with survival outcomes and subsequent maintenance strategies among patients who achieved initial benefit from T-DXd.

## 2. Materials and Methods

### 2.1. Study Design and Patient Population

This was a single-center, retrospective, real-world study conducted at the Department of Breast Oncology, Peking University Cancer Hospital. Consecutive patients with metastatic breast cancer who received T-DXd between March 2023 and March 2026 were screened from institutional clinical databases.

Patients were eligible if they met the following criteria: (1) histologically confirmed metastatic breast cancer; (2) HER2-positive disease, defined as an immunohistochemistry (IHC) score of 3+ or an IHC score of 2+ with positive fluorescence in situ hybridization (FISH), based on the most recent available tumor specimen from either the primary or metastatic site; (3) receipt of at least one dose of T-DXd; and (4) at least one radiological tumor assessment following T-DXd initiation.

The study was approved by the institutional review board of Peking University Cancer Hospital (approval code: 2025YJZ40; approval date: 11 April 2025). This work was supported by the Science Foundation of Peking University Cancer Hospital ZY202510.

### 2.2. Data Collection and Baseline Variables

Demographic, clinicopathologic, and treatment-related data were extracted from electronic medical records. Collected variables included age, sex, body mass index (BMI), Eastern Cooperative Oncology Group (ECOG) performance status, hormone receptor (HR) status, HER2 IHC score, de novo metastatic status, histologic subtype, metastatic sites (visceral, liver, lung, and CNS), prior systemic therapies, number of prior treatment lines in the metastatic setting, prior anti-HER2 treatment exposure, and T-DXd treatment characteristics. HR positivity was defined as ≥1% nuclear staining for estrogen receptor and/or progesterone receptor.

T-DXd (trastuzumab deruxtecan [ENHERTU^®^]; Baxter Oncology GmbH, Halle/Westfalen, Germany) was administered intravenously according to routine clinical practice, with a recommended dose of 5.4 mg/kg every three weeks. Alternative starting doses or dose reductions were permitted at the treating physician’s discretion.

### 2.3. Efficacy and Safety Assessments

Tumor response was assessed radiographically according to routine clinical practice, generally based on the Response Evaluation Criteria in Solid Tumors (RECIST), version 1.1. The best overall response was classified as complete response (CR), partial response (PR), stable disease (SD), or progressive disease (PD). Objective response rate (ORR) was defined as the proportion of patients achieving CR or PR and was calculated among patients with measurable disease. For descriptive best-response analysis, patients without measurable lesions were included when follow-up imaging was available. Those without documented progression were categorized as non-CR/non-PD.

Progression-free survival (PFS) was defined as the time from initiation of T-DXd to documented disease progression or death from any cause, whichever occurred first. Overall survival (OS) was defined as the time from T-DXd initiation to death from any cause. Patients without an event were censored at the date of last follow-up. Brain imaging studies were retrospectively reviewed by investigators, and intracranial response was assessed according to RECIST criteria based on radiological evaluation. CNS-directed local therapy administered within 1 month before T-DXd initiation or during T-DXd treatment was defined as peri-T-DXd CNS local therapy. CNS local treatment included radiotherapy and neurosurgical intervention. Active CNS metastases were defined as radiographically evident untreated or progressing CNS disease at the time of T-DXd initiation. CNS-ORR was defined as the proportion of evaluable patients with CNS metastases who achieved CNS CR or PR. Primary resistance to T-DXd was defined as radiologic PD at the first efficacy assessment after treatment initiation.

Adverse events were collected from medical records and graded according to the National Cancer Institute Common Terminology Criteria for Adverse Events (CTCAE), version 5.0.

### 2.4. Statistical Analysis

Descriptive statistics were used to summarize baseline characteristics. Continuous variables are reported as medians with ranges or means with standard deviations, as appropriate, and categorical variables are reported as counts and percentages. Group comparisons were performed using the chi-square test or Fisher’s exact test for categorical variables and the independent-sample *t*-test or Mann–Whitney U test for continuous variables, as appropriate.

PFS and OS were estimated using the Kaplan–Meier method, and differences between groups were compared using the log-rank test. Cox proportional hazards regression models were used to identify factors associated with PFS and OS. Clinically relevant and/or univariably significant variables were included in multivariable models. Results are reported as hazard ratios (HRs) with 95% confidence intervals (CIs). Because the best overall response was a post-baseline variable, it was not included as a baseline covariate in the Cox regression models.

All statistical analyses were performed using SPSS version 26.0, R version 4.6.0, and GraphPad Prism 10. All tests were two-sided, and *p* < 0.05 was considered statistically significant.

## 3. Results

### 3.1. Patient Characteristics

Among 199 patients treated with T-DXd, 173 were eligible for analysis (Figure S1). The remaining 26 patients were excluded due to the absence of post-treatment radiological assessment, including terminal disease status (*n* = 5), financial considerations (*n* = 5), no follow-up imaging (*n* = 8), adverse events (*n* = 4), and personal choice (*n* = 4). The median age was 57.6 years, and the median BMI was 24.1 kg/m^2^. Visceral metastases were present in 147 patients (85.0%), and 63 patients (36.4%) had active CNS metastases. The median number of prior overall treatment lines in the metastatic setting was two. All patients had received prior anti-HER2 monoclonal antibody therapy with trastuzumab and/or inetetamab. Prior exposure rate to TKIs was 80.3% (Table 1).

### 3.2. Efficacy

**Figure 1:**
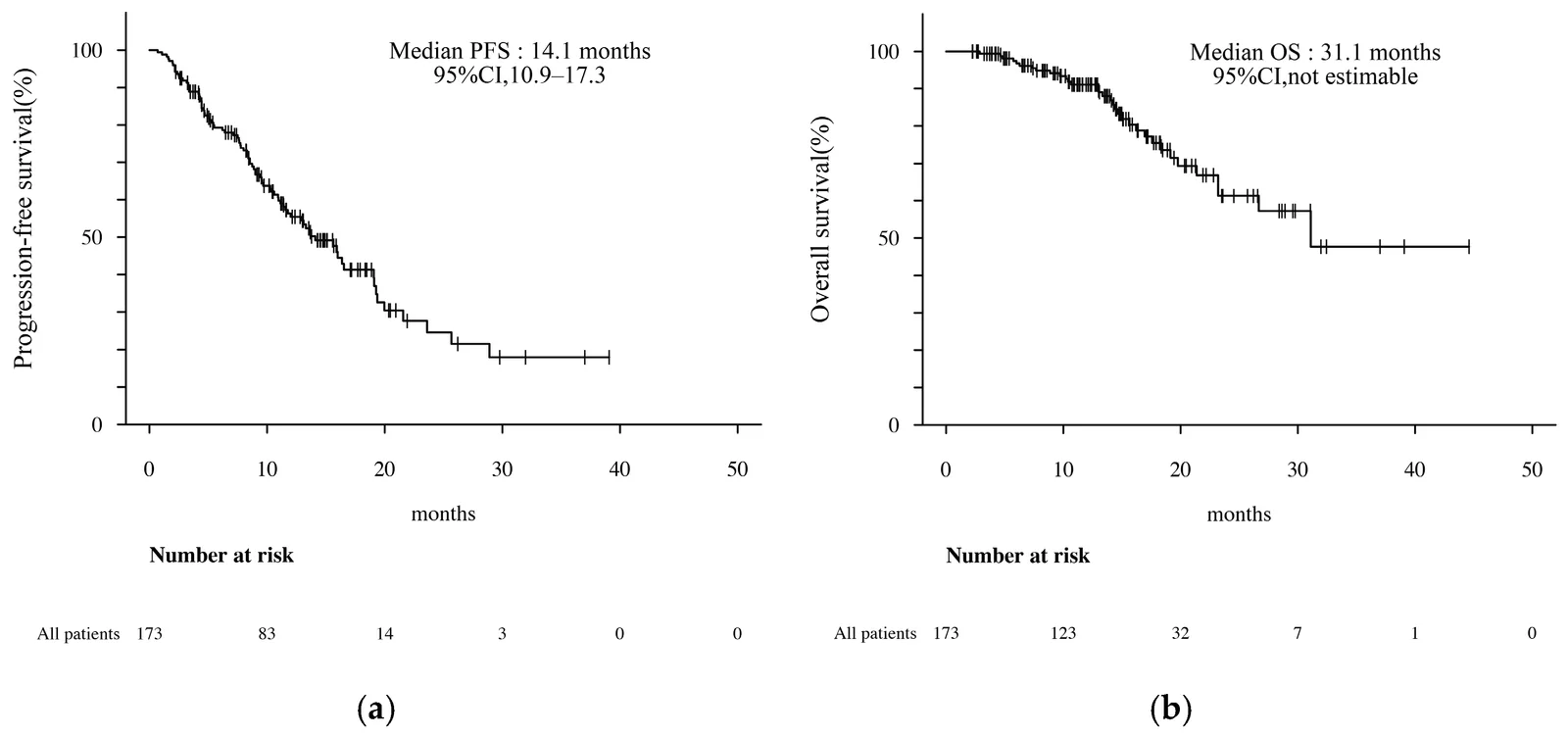
Survival outcomes in the overall cohort. The Kaplan–Meier curve for median PFS of the entire cohort (**a**); the Kaplan–Meier curve for median OS of the entire cohort (**b**). PFS, progression-free survival; OS, overall survival.

After a median follow-up of 14.3 months, median progression-free survival (PFS) was 14.1 months, and median overall survival (OS) was 31.1 months (Figure 1). Among the 154 patients with measurable systemic disease, the ORR was 54.5%. Primary resistance to T-DXd was observed in 9 of the overall 173 patients. Among the 72 patients with CNS metastases, 14 underwent radiotherapy or CNS surgery during the peri-T-DXd period, including surgical resection of brain metastases in 6 patients and radiotherapy (whole-brain radiotherapy or stereotactic radiosurgery) in 8 patients. Among the 67 patients with measurable CNS lesions, the CNS objective response rate (CNS-ORR) was 46.3%, including 4 complete CNS responses. Among the 63 patients with active CNS metastases, the CNS-ORR was 47.6%. Primary CNS resistance was observed in 2 patients. We further performed a stratified analysis according to peri-T-DXd CNS-directed local treatment. Among patients with measurable CNS lesions, the CNS-ORR was comparable between patients with and without peri-T-DXd local treatment (53.8% vs. 44.4%, *p* = 0.21). Of the four patients who achieved complete CNS response, three did not receive peri-T-DXd CNS-directed local treatment, whereas one received local treatment.

Subgroup analyses are summarized in Table S1. No significant differences in PFS were observed with respect to age, BMI, hormone receptor status, or the presence of visceral, liver, lung, or CNS metastases. Patients with HER2 IHC 3+ tumors showed a numerically longer median PFS than those with HER2 IHC 2+/FISH-positive tumors (15.6 vs. 10.9 months, *p* = 0.09).

By contrast, greater prior treatment exposure was associated with shorter PFS. Patients who had received ≥3 prior chemotherapy lines in the metastatic setting had a significantly shorter median PFS than those who had received <3 prior lines (11.6 vs. 19.1 months, *p* = 0.006). Similarly, patients who had received ≥3 prior anti-HER2 treatment lines had a significantly shorter median PFS than those who had received <3 such lines (11.8 vs. 19.1 months, *p* = 0.02). In an additional analysis stratified by 0–1 versus >1 prior anti-HER2 treatment lines, median PFS was 23.6 and 13.6 months, respectively (*p* = 0.10).

Patients with prior TKI exposure had received more previous treatment lines than TKI-naïve patients (*p* < 0.01; Table S2). However, prior TKI exposure was not associated with significantly different PFS (13.7 vs. 23.6 months; *p* = 0.34) or OS (31.0 months vs. not reached; *p* = 0.34).

Patients who achieved a partial response had markedly longer PFS than those with stable disease (23.6 vs. 9.6 months, *p* < 0.001), and their OS was also longer (not reached vs. 19.8 months, *p* < 0.001). In addition, patients who initiated T-DXd at a starting dose of ≥4.4 mg/kg had significantly longer PFS than those who started at <4.4 mg/kg (16.5 vs. 11.0 months, *p* = 0.01). Neither maintenance dose nor weight change during treatment was significantly associated with PFS or OS (Figure S2).

### 3.3. Univariable and Multivariable Cox Regression Analysis

**Figure 2:**
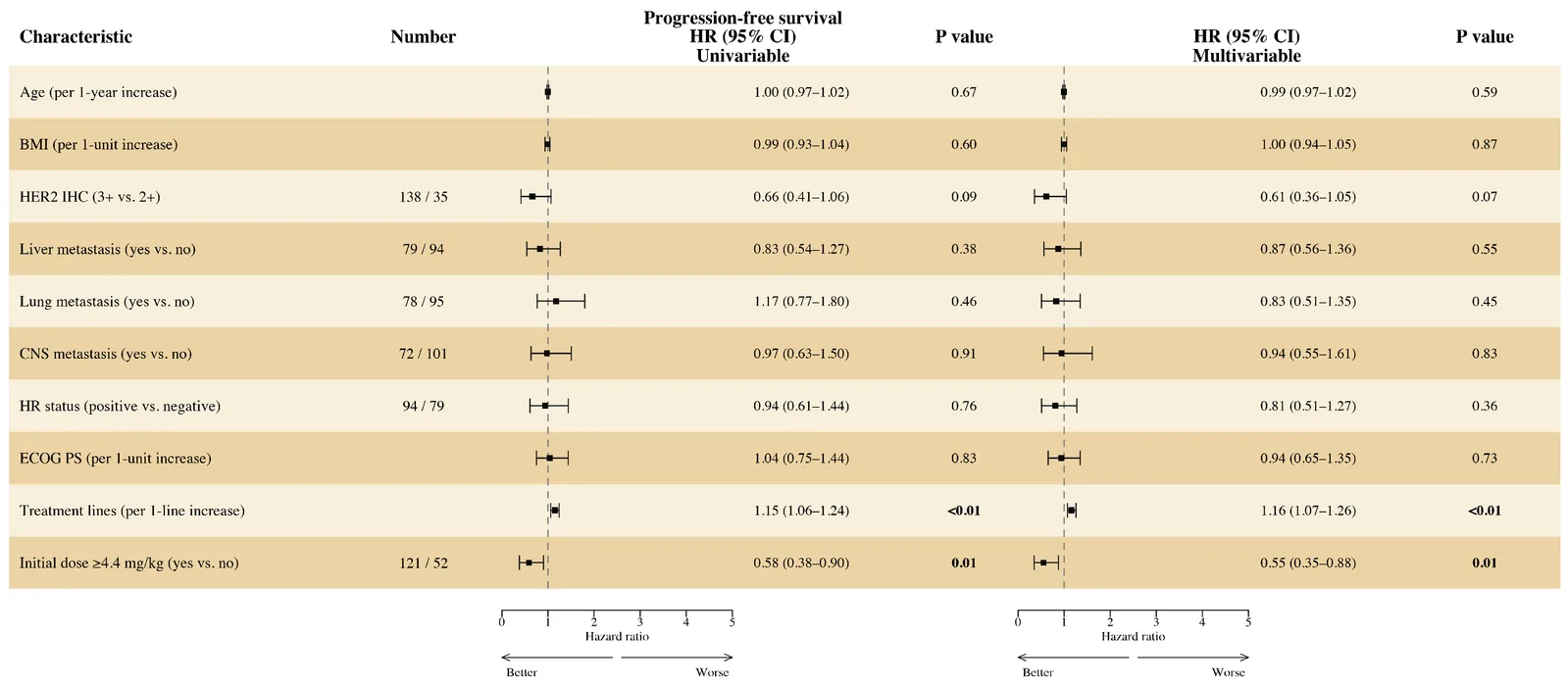
Univariable and multivariable Cox regression analysis for progression-free survival. BMI, body mass index; CI, confidence interval; CNS, central nervous system; ECOG, Eastern Cooperative Oncology Group; HER2, human epidermal growth factor receptor 2; HR status, hormone receptor status; HR, hazard ratio; IHC, immunohistochemistry; PFS, progression-free survival. The dashed line represents HR = 1.

**Figure 3:**
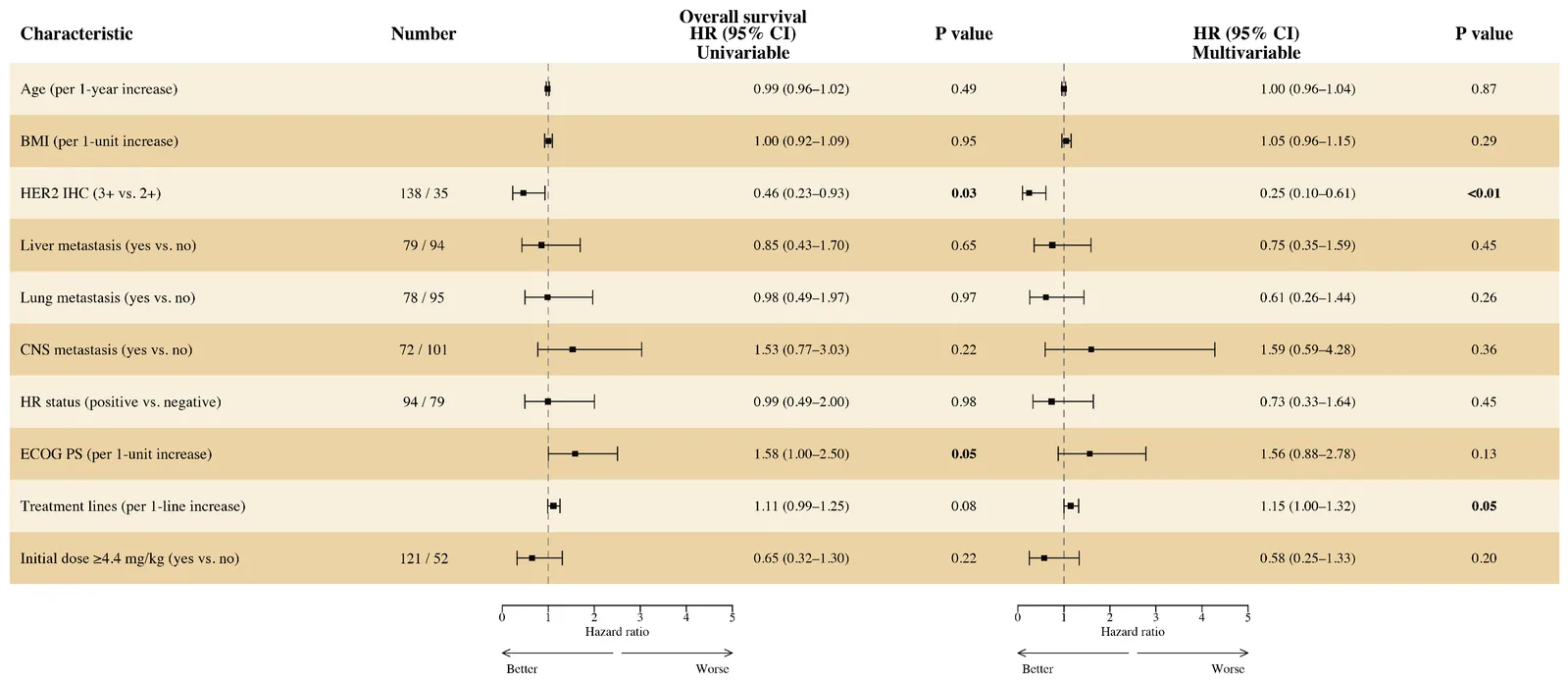
Univariable and multivariable Cox regression analysis for overall survival. BMI, body mass index; CI, confidence interval; CNS, central nervous system; ECOG, Eastern Cooperative Oncology Group; HER2, human epidermal growth factor receptor 2; HR status, hormone receptor status; HR, hazard ratio; IHC, immunohistochemistry; OS, overall survival. The dashed line represents HR = 1.

Univariable and multivariable Cox regression analyses consistently showed that a greater number of prior treatment lines was associated with worse PFS, whereas an initial dose ≥ 4.4 mg/kg was associated with improved PFS. HER2 IHC 3+ status was independently associated with longer OS and showed a nonsignificant trend toward improved PFS. No other baseline factors were consistently associated with survival outcomes (Figure 2 and Figure 3).

### 3.4. Exploratory Analysis of Subsequent Treatment Course After Initial Benefit from T-DXd

After restricting the analysis to patients with at least five treatment cycles, 148 patients were included in the comparison of subsequent treatment strategies. Of these, 125 continued T-DXd until disease progression, whereas 23 switched to an alternative maintenance regimen. Ten patients switched to pyrotinib, nine switched to monoclonal antibody-based therapy, and four switched to trastuzumab rezetecan. Patients in the switch-to-maintenance group received a median of 5 cycles of T-DXd treatment. Reasons for switching to maintenance therapy included sustained tumor control without further shrinkage, adverse events (including interstitial lung disease (ILD), fatigue, gastrointestinal symptoms, and cardiovascular symptoms), financial considerations, and patient preference.

Using PFS measured from T-DXd initiation to the first documented disease progression or death from any cause, no statistically significant difference in PFS or OS was observed between the switch-to-maintenance group and the T-DXd continuation group (median PFS, not reached vs. 15.0 months, *p* = 0.07; median OS, not reached vs. 31.1 months, *p* = 0.14).

Among the 23 patients who switched to maintenance therapy, 15 achieved a partial response, and 8 had stable disease before the treatment switch. Ten patients in this group had CNS metastases, of whom nine achieved a CNS response.

Baseline characteristics were generally balanced between the two groups. The median age was 56.7 years in the T-DXd continuation group and 62.2 years in the switch-to-maintenance group (*p* = 0.18). No significant between-group differences were observed in BMI, ECOG performance status, hormone receptor status, HER2 IHC status, visceral metastases, liver metastases, lung metastases, CNS metastases, active CNS metastases, number of prior treatment lines, or number of prior anti-HER2 treatment lines. The only statistically significant imbalance was prior pertuzumab exposure, which was less frequent in the switch-to-maintenance group than in the T-DXd continuation group (56.5% vs. 85.6%, *p* = 0.003) (Table 2).

### 3.5. Safety

The most common adverse events were fatigue (59.5%), nausea (58.4%), leukopenia (50.9%), and neutropenia (48.6%). Grade ≥ 3 adverse events mainly included neutropenia (9.2%), fatigue (5.2%), leukopenia (5.2%), nausea (4.0%), and thrombocytopenia (2.3%) (Table 3).

ILD occurred in 13 patients (7.5%), and all cases were grade 1–2, including 9 grade 1 events and 4 grade 2 events. The median cycle at ILD onset was 5 (range, 2–16). Three patients with grade 2 ILD discontinued T-DXd, and one patient with grade 2 ILD resumed T-DXd at a reduced dose after recovery from ILD.

No patient discontinued treatment or underwent dose reduction because of a decline in left ventricular ejection fraction (LVEF) on echocardiography. Cardiac-related event monitoring abnormalities were uncommon and included QT interval prolongation in 1 patient, palpitations in 3 patients, and elevated myocardial injury biomarkers in 2 patients. One patient was found to have mitral valve prolapse on echocardiography, which led to T-DXd discontinuation. However, its causal relationship with T-DXd was not established.

The median initial dose in this study population was 4.7 mg/kg, and the median maintenance dose was 4.5 mg/kg. Dose reduction occurred in 34 cases, of which 32 were attributed to adverse events. The most common reasons were gastrointestinal symptoms (*n* = 12), fatigue (*n* = 9), palpitations or abnormal cardiac biomarkers (*n* = 4), myelosuppression (*n* = 4), and ILD (*n* = 3).

## 4. Discussion

To our knowledge, this is one of the largest single-center real-world studies to evaluate T-DXd in patients with HER2-positive advanced breast cancer. Our findings support the systemic and intracranial activity and manageable safety of T-DXd in routine clinical practice. This study also extends existing evidence by exploring subsequent maintenance strategies among patients who derived initial benefit from T-DXd, a scenario that remains insufficiently characterized.

The efficacy observed in our cohort was generally consistent with, although numerically lower than, that reported in pivotal clinical trials [1,9]. Nearly half of our patients had received three or more prior lines, reflecting a heavily pretreated population. Consistently, greater prior treatment exposure was associated with inferior outcomes [6]. These findings support earlier use of T-DXd before the emergence of complex treatment resistance, a strategy further supported by DESTINY-Breast09 [2] and the subsequent U.S. FDA [10] approval of first-line T-DXd plus pertuzumab in December 2025.

In our cohort, HER2 IHC 3+ disease was associated with significantly longer OS (*p* < 0.01) and numerically longer PFS than HER2 IHC 2+/FISH-positive disease. This is consistent with real-world evidence [6] showing better T-DXd outcomes in tumors with higher HER2 expression, including observations in HER2-low breast cancer that IHC 2+ tumors may derive greater benefit than IHC 1+ tumors [11]. Paired-sample analyses have shown that lower baseline HER2 IHC expression is associated with a higher likelihood of HER2 loss after T-DXd [12], and studies in other anti-HER2 settings have consistently demonstrated greater benefit in IHC 3+ than in IHC 2+/FISH-positive tumors [11,13,14]. Together, these findings suggest that HER2 protein expression levels may remain clinically relevant even within conventionally defined HER2-positive disease. Given the potential for treatment-induced HER2 changes and spatial heterogeneity, re-biopsy may be valuable when feasible after disease progression.

Another important finding was the intracranial activity of T-DXd in a real-world population with a high CNS disease burden and extensive prior exposure to HER2-directed TKIs. CNS metastases were present in 41.6% of patients, including active CNS metastases in 36.4%. This proportion was comparable to that reported in a previous Chinese cohort [6] (38.7%) but higher than that in the DE-REAL study [8] (25%). Before national reimbursement took effect in January 2025, substantial out-of-pocket costs may have led clinicians and patients to preferentially consider T-DXd for those with a greater unmet clinical need, including patients with CNS involvement, given its emerging intracranial activity.

Among patients with measurable CNS lesions, the CNS-ORR was 46.3%, and among those with active CNS metastases, it was 47.6%. Among patients without peri-T-DXd local treatment, the CNS-ORR was 44.4%. Unlike several prospective or real-world cohorts [3,4,15] in which prior HER2-directed TKI exposure was limited, more than 80% of our patients had previously received TKIs. This reflects the treatment landscape in China, where pyrotinib has been widely used for HER2-positive metastatic breast cancer with CNS metastases, supported by the intracranial antitumor activity observed in the PERMEATE study [16]. Thus, although our CNS-ORR was numerically lower than that reported in previous studies, it remains clinically relevant and suggests that T-DXd may retain intracranial efficacy after prior TKI-based therapy [17].

Recent clinical and real-world studies have further strengthened the evidence supporting the CNS activity of T-DXd. In the phase 3b/4 DESTINY-Breast12 study [4], T-DXd demonstrated clinically meaningful intracranial responses in patients with HER2-positive metastatic breast cancer with brain metastases, including patients with active CNS disease. Real-world evidence has subsequently extended these findings to routine clinical practice. In an Italian multicenter real-world analysis focusing specifically on patients with HER2-positive breast cancer brain metastases, Fabi et al. [18] reported an intracranial ORR of 59%, an intracranial disease control rate of 94.9%, and a median intracranial PFS of 15.6 months, confirming substantial intracranial activity outside clinical trials. Similarly, Duan et al. [19] evaluated patients with active brain metastases from HER2-positive/HER2-low metastatic breast cancer and reported an intracranial ORR of 65.5% among HER2-positive patients using RANO-BM criteria. However, these cohorts were characterized by smaller sample sizes and selected patient populations, including specific inclusion criteria for measurable or active CNS lesions. Differences in prior treatment exposure, CNS disease characteristics, and patient selection may partly explain the numerical differences in CNS response rates across studies.

The potential role of local CNS-directed treatment during T-DXd therapy remains an area of clinical uncertainty. In our study, peri-T-DXd local treatment was defined as radiotherapy or surgery performed within one month before T-DXd initiation or during T-DXd treatment. Among patients receiving peri-T-DXd local treatment, the CNS-ORR was numerically higher than that among patients without peri-T-DXd local treatment (53.8% vs. 44.4%). However, these findings should not be interpreted as evidence of a synergistic effect between local therapy and T-DXd, because decisions regarding CNS-directed treatment were made according to physician preference and individual clinical characteristics. Patients selected for local therapy may have had more symptomatic, larger, or rapidly progressive CNS lesions, introducing potential confounding by indication. Therefore, the optimal sequencing and combination strategy of T-DXd with CNS-directed local therapy remains unclear.

Although T-DXd contains a cytotoxic payload, raising theoretical concerns regarding concurrent administration with radiotherapy, emerging real-world evidence suggests that this combination may be feasible in selected patients. The TENDANCE multicentric French study [20] and a large international real-world cohort [21] reported that concurrent use of T-DXd and radiotherapy, including CNS-directed radiotherapy, was generally feasible without excessive severe toxicity. Similarly, single-center experiences [22] and expert reviews [23] have supported cautious use of T-DXd with radiotherapy in clinical practice. However, these studies were retrospective and heterogeneous regarding radiotherapy indications, timing, dose, and treated sites. Moreover, they primarily addressed safety rather than comparative intracranial efficacy. Prospective studies are needed to determine whether concurrent or sequential CNS-directed therapy can optimize intracranial control while maintaining the safety profile of T-DXd.

A distinctive aspect of our study is the exploratory analysis of patients who initially benefited from T-DXd and subsequently switched to alternative maintenance therapy. In our cohort, 23 patients (13.3%) switched to alternative maintenance regimens after initial T-DXd benefit, mainly due to sustained disease control without further tumor shrinkage, adverse events, financial considerations, or patient preference. After restricting the analysis to patients who remained alive and progression-free after approximately five cycles of T-DXd treatment, no statistically significant difference in PFS or OS was observed between the switch-to-maintenance and T-DXd-continuation groups, although PFS numerically favored continued T-DXd. Therefore, these findings should not be interpreted as evidence of comparable efficacy between treatment strategies. Given the retrospective design, limited sample size, heterogeneous reasons for treatment transition, patient selection, and potential immortal time-related bias, this exploratory analysis should be interpreted cautiously and considered hypothesis-generating.

Treatment adaptation strategies may become increasingly relevant in HER2-positive metastatic breast cancer as patients achieve prolonged disease control with effective HER2-targeted therapies. Similar clinical scenarios have emerged as T-DXd moves into earlier treatment lines and produces more prolonged disease control, including treatment discontinuation without progression and subsequent maintenance therapy in DESTINY-Breast09 [2]. Although the PATINA trial [24] evaluated a different maintenance strategy involving CDK4/6 inhibition plus endocrine therapy after induction chemotherapy and anti-HER2 therapy in HR-positive/HER2-positive metastatic breast cancer, it provides a conceptual framework supporting further investigation of maintenance approaches in this disease setting. Although our study provides preliminary real-world observations regarding treatment transition after T-DXd benefit, current evidence remains insufficient to support routine switching from T-DXd to maintenance therapy. Prospective studies with predefined treatment strategies, appropriate statistical designs, and longer follow-up are required to determine whether treatment de-escalation or maintenance approaches can provide comparable disease control while improving tolerability, quality of life, or treatment feasibility.

In our study, patients achieving PR had a longer PFS than those with SD (23.6 vs. 9.6 months), suggesting that deeper tumor shrinkage was associated with more durable benefits. This observation is consistent with exploratory analyses from the phase III DESTINY-Breast09 study presented at the 2026 ASCO Annual Meeting, which reported an association between greater depth of response and longer PFS among patients receiving first-line T-DXd-based therapy [25]. However, because best response is a post-treatment variable, this finding should be interpreted as a marker of treatment benefit rather than a baseline prognostic factor.

We also observed an association between an initial dose of ≥4.4 mg/kg and longer PFS. However, this finding should be interpreted with caution, as the choice of initial dose in real-world practice is often influenced by patient characteristics, including frailty, comorbidities, ECOG performance status, and anticipated treatment tolerance. Patients receiving reduced initial doses may represent a more vulnerable population with poorer baseline conditions, although this could not be fully demonstrated in our cohort. These underlying factors may themselves be associated with shorter PFS. Therefore, the observed association between initial dose and PFS may be partly affected by confounding by indication rather than representing a direct dose–efficacy relationship. In contrast, maintained dose was not significantly associated with survival. Consistently, a retrospective study [26] from Singapore reported that an initial relative dose intensity of T-DXd below 85% did not significantly compromise rwPFS in patients with metastatic breast cancer. Given the financial burden of T-DXd and the potential for adverse events, individualized dose adjustment is often unavoidable in real-world practice, and further evidence is needed to better define its clinical impact.

The safety profile observed in our cohort was manageable and broadly consistent with previous studies. The incidence of grade ≥ 3 adverse events appeared lower than that reported in clinical trials, possibly owing to more proactive supportive care in real-world practice, such as prophylactic use of granulocyte colony-stimulating factors and lower actual dose intensity (4.5 mg/kg). ILD remains a clinically relevant toxicity associated with T-DXd. In our cohort, ILD occurred in 13 patients (7.5%), and all events were grade 1–2, with no grade ≥ 3 ILD observed. This incidence appeared lower than that reported in pivotal clinical trials, such as DESTINY-Breast02 and DESTINY-Breast03, in which ILD was reported in approximately 10–15% of patients. However, ILD incidence has varied substantially across real-world studies. For example, the DE-REAL study reported an ILD incidence of approximately 2%, whereas a large real-world study by Xue et al. reported ILD in 7.5% of patients with HER2-positive disease, including 4.6% grade 3–4 events. Overall, the incidence of ILD observed in routine practice appears variable and may be influenced by real-world dose modifications, differences in surveillance intensity and imaging practices, and the retrospective nature of adverse event collection. Although no treatment discontinuation due to decreased LVEF was observed, one patient developed mitral valve prolapse on echocardiography, and two patients had elevated myocardial injury biomarkers. These events have been uncommon in previous reports and warrant further evaluation in larger real-world cohorts.

Our study has several limitations. First, this was a retrospective, single-center study including only Chinese patients, and treatment decisions, imaging intervals, response assessments, dose modifications, and maintenance strategies were not protocol-defined. Selection bias could not be completely avoided. Second, the maintenance analysis was limited by small sample size, treatment heterogeneity, patient selection, and potential time-dependent and immortal time biases because patients who switched to maintenance therapy had already achieved initial disease control and survived long enough to undergo treatment transition. Third, genomic data were unavailable, precluding further exploration of molecular predictors of T-DXd efficacy or resistance. Finally, treatment sequencing in this cohort may differ from that in international trial populations, partly because T-DM1 became broadly accessible later in China after reimbursement approval. Prospective multicenter studies incorporating standardized response assessment, predefined dose-modification and maintenance strategies, and integrated genomic profiling are needed to validate these findings.

## 5. Conclusions

This single-center, real-world study supports the systemic and intracranial activity and manageable safety profile of T-DXd in HER2-positive advanced breast cancer, including patients with active CNS metastases and extensive prior HER2-directed TKI exposure. The exploratory analysis of subsequent treatment strategies was subject to potential selection and immortal time-related biases, precluding conclusions regarding the comparative effectiveness of maintenance strategies. These findings are hypothesis-generating and require prospective validation.

## Figures and Tables

**Table 1:** Baseline demographic and clinical characteristics.

	Patients, *n* (%)
Median age (range) yr	57.6 (32.7–80.0)
Female sex, *n* (%)	173 (100.0)
Median BMI (range)	24.1 (15.6–40.2)
ECOG	
0	109 (63.0)
≥1	64 (37.0)
De novo metastatic disease, *n* (%)	49 (28.3)
Hormone receptor status, *n* (%)	
Positive	94 (54.3)
Negative	79 (45.7)
Histology, *n* (%)	
Invasive ductal carcinoma	149 (86.1)
Invasive lobular carcinoma	4 (2.3)
Micropapillary carcinoma	11 (6.4)
Others	9 (5.2)
HER2 status, *n* (%)	
IHC 2+/FISH-positive	35 (20.2)
IHC 3+	138 (79.8)
Visceral metastases, *n* (%)	147 (85.0)
Liver metastases	79 (45.7)
Lung metastases	78 (45.1)
CNS metastases	72 (41.6)
Active CNS metastases, *n* (%)	63 (36.4)
Lines of previous therapy for metastatic disease	
Median no. of lines (range)	2 (1–16)
1	41 (23.7)
2	50 (28.9)
≥3	82 (47.4)
Lines of previous anti-HER2 therapy for metastatic disease	
Median no. of lines (range)	2 (0–16)
0–1	45 (26.0)
2	53 (30.6)
≥3	75 (43.4)
Lines of previous chemotherapy for metastatic disease	
Median no. of lines (range)	2 (0–16)
0–1	46 (26.6)
2	52 (30.0)
≥3	75 (43.4)
Prior anti-HER2 targeted therapy in the metastatic setting, *n* (%)	
Trastuzumab	159 (91.9)
Pertuzumab	137 (79.2)
T-DM1	48 (27.7)
TKI	139 (80.3)
Inetetamab	79 (45.7)
Other anti-HER2 ADCs	16 (9.2)

BMI, body mass index; ECOG, Eastern Cooperative Oncology Group performance status; IHC, immunohistochemistry; FISH, fluorescence in situ hybridization; CNS, central nervous system; TKI, tyrosine kinase inhibitor; T-DM1, trastuzumab emtansine; ADC, antibody–drug conjugate.

**Table 2:** Characteristics of patients in the T-DXd continuation group and the switch-to-maintenance group.

	T-DXd Continuation Group (*n* = 125)	Switch-to-Maintenance Group (*n* = 23)	*p*
Median age (range) yr	56.7 (34.0–80.0)	62.2 (32.7–75.9)	0.18
Median BMI (range)	23.6 (15.6–35.7)	24.4 (18.4–38.1)	0.35
ECOG			0.82
0	79 (63.2)	14 (60.9)	
≥1	46 (36.8)	9 (39.1)	
Hormone receptor status, *n* (%)			0.26
Positive	71 (56.8)	10 (43.5)	
Negative	54 (43.2)	13 (56.5)	
HER2 status, *n* (%)			0.77
IHC 2+/FISH-positive	22 (17.6)	3 (13.0)	
IHC 3+	103 (82.4)	20 (87.0)	
Visceral metastases, *n* (%)	109 (87.2)	21 (91.3)	0.74
Liver metastases	56 (44.8)	10 (43.5)	1.00
Lung metastases	60 (48.0)	9 (39.1)	0.50
CNS metastases	52 (41.6)	10 (43.5)	1.00
Active CNS metastases, *n* (%)	48 (38.4)	8 (34.8)	0.64
Lines of previous therapy for metastatic disease, median no. of lines (range)	2 (1–16)	2 (1–7)	0.41
1, *n* (%)	31 (24.8)	7 (30.4)	
2, *n* (%)	32 (25.6)	6 (26.1)	
≥3, *n* (%)	62 (49.6)	10 (43.5)	
Prior anti-HER2 targeted therapy in the metastatic setting, *n* (%)			
Trastuzumab	118 (94.4)	20 (87.0)	0.19
Pertuzumab	107 (85.6)	13 (56.5)	<0.01
T-DM1	36 (28.8)	3 (13.0)	0.13
TKI	101 (80.8)	18 (78.3)	0.78
Inetetamab	54 (43.2)	10 (43.5)	1.00
Other anti-HER2 ADCs	10 (8.0)	1 (4.3)	1.00
Initial dose			0.47
≥4.4 mg/kg	87 (69.6)	14 (60.9)	
<4.4 mg/kg	38 (30.4)	9 (39.1)	

Patients were categorized into the T-DXd continuation group and the switch-to-maintenance group according to whether T-DXd was continued until progression or replaced by an alternative maintenance regimen before progression. This analysis included 148 patients with at least 5 cycles of T-DXd. BMI, body mass index; CNS, central nervous system; ECOG, Eastern Cooperative Oncology Group; FISH, fluorescence in situ hybridization; HR, hormone receptor; IHC, immunohistochemistry; T-DM1, trastuzumab emtansine; T-DXd, trastuzumab deruxtecan; TKI, tyrosine kinase inhibitor; ADC, antibody–drug conjugate.

**Table 3:** Summary of adverse events.

Adverse Event	All Grades, No. (%)	Grade ≥ 3, No. (%)
Fatigue	103 (59.5)	9 (5.2)
Nausea	101 (58.4)	7 (4.0)
Leukopenia	88 (50.9)	9 (5.2)
Neutropenia	84 (48.6)	16 (9.2)
Hepatic laboratory abnormalities	57 (32.9)	0 (0.0)
Alopecia	35 (20.2)	1 (0.6)
Anemia	33 (19.1)	1 (0.6)
Thrombocytopenia	25 (14.5)	4 (2.3)
Constipation	22 (12.7)	0 (0.0)
Vomiting	19 (11.0)	2 (1.2)
Interstitial lung disease	13 (7.5)	0 (0.0)
Creatine kinase elevation	7 (4.0)	0 (0.0)
Cardiac monitoring abnormalities	6 (3.5)	0 (0.0)

Hepatic laboratory abnormalities referred to elevations in liver enzymes and/or bilirubin levels identified during treatment. Cardiac monitoring abnormalities included palpitations in 3 patients, elevated myocardial injury biomarkers in 2 patients and QT interval prolongation in 1 patient. AE, adverse event.

## Data Availability

De-identified data supporting the findings of this study may be made available from the corresponding authors upon reasonable request, subject to institutional approval and applicable data protection regulations.

## Supplementary Materials

The following supporting information can be downloaded at https://www.mdpi.com/article/10.3390/curroncol33080487/s1, Table S1: Baseline characteristics and survival analysis; Table S2: Baseline characteristics by prior anti-HER2 TKI exposure; Figure S1: Patient selection flow diagram for the final analysis cohort; Figure S2: Survival outcomes of patients in clinically relevant subgroups.
