# Supplementary material for: Trastuzumab Deruxtecan in HER2-Positive Metastatic Breast Cancer: Real-World Outcomes and Treatment Transition Patterns in a Chinese Cohort
Source: Curr Oncol. 2026 Aug 18;33(8):487. doi: 10.3390/curroncol33080487 (PMC13510711; doi:10.3390/curroncol33080487)
Supplement: Supplementary file 1 [file curroncol-33-00487-s001.zip › Supplemental Tables.pdf]

Table S1. Baseline characteristics and survival analysis

Characteristic	No. (%)	PFS, months	P value	OS, months	P value
Total	173 (100.0)	14.1		31.1	
Age, years			0.30		0.27
<65	130 (75.1)	13.7		31.1	
≥65	43 (24.9)	15.6		NR	
BMI, kg/m²			0.88		0.84
<25	106 (61.3)	15.6		31.1	
≥25	67 (38.7)	13.7		NR	
HR status			0.77		0.98
Negative	79 (45.7)	13.7		NR	
Positive	94 (54.3)	14.1		31.1	
HER2 IHC status			0.09		0.03
2+	35 (20.2)	10.9		21.4	
3+	138 (79.8)	15.6		NR	
Visceral metastases			0.13		0.61
Yes	147 (85.0)	15.6		31.1	
No	26 (15.0)	9.6		NR	
Liver metastases			0.38		0.64
Yes	79 (45.7)	15.9		31.1	
No	94 (54.3)	12.0		NR	
Lung metastases			0.46		0.97
Yes	78 (45.1)	13.1		31.1	
No	95 (54.9)	16.0		NR	
CNS metastases			0.90		0.22
Yes	72 (41.6)	15.6		26.7	
No	101 (58.4)	13.3		NR	
Lines of previous chemotherapy for metastatic disease			<0.01		0.32
< 3	98 (56.6)	19.1		NR	
≥3	75 (43.4)	11.6		26.7	
Lines of previous anti-HER2 therapy for metastatic disease			0.02		0.23
< 3	98 (56.6)	19.1		NR	
≥3	75 (43.4)	11.8		26.7	
Lines of previous anti-HER2 therapy for metastatic disease			0.10		0.51
≤1	45	23.6		NR	
>1	128	13.6		31.1	
Weight change			0.83		0.48
Weight loss	82 (47.4)	13.6		31.1	
Maintain or gain weight	91 (52.6)	15.6		NR	
Initial dose			0.01		0.22
≥4.4 mg/kg	121 (69.9)	16.5		31.1	
<4.4 mg/kg	52 (30.1)	11.0		NR	
Maintained dose			0.68		0.31
≥4.4 mg/kg	98 (56.6)	15.9		NR	
<4.4 mg/kg	75 (43.4)	13.1		31.1	

Best response			<0.01		<0.01
SD	80 (48.8)	9.6		19.8	
PR	84 (51.2)	23.6		NR	

CNS, central nervous system; OS, overall survival; PFS, progression-free survival; SD, stable disease; PR, partial response; NR,not reached. Best response analysis was performed among 164 patients after excluding 9 patients with primary progressive disease. Patients with measurable disease were assessed according to RECIST-based radiological evaluation. Patients without measurable lesions who had no documented disease progression during follow-up were categorized as non-CR/non-PD and included in the descriptive SD group.

Table S2. Baseline characteristics by prior anti-HER2 TKI exposure

**Characteristic**	**TKI-pretreated** (n=139)	**TKI-naïve** (n=34)	**P value**
Median age (range), yr	57.7 (32.7-75.6)	56.9 (34.0-80.0)	0.40
Median BMI (range)	24.1 (15.6-40.2)	23.8 (16.8-30.8)	0.71
ECOG, n (%)			0.08
0	83 (59.7)	26 (76.5)	
≥1	56 (40.3)	8 (23.5)	
Hormone receptor status, n (%)			1.00
Positive	75 (54.0)	19 (55.9)	
Negative	64 (46.0)	15 (44.1)	
HER2 status, n (%)			0.34
IHC 2+/FISH-positive	26 (18.7)	9 (26.5)	
IHC 3+	113 (81.3)	25 (73.5)	
Visceral metastases, n (%)	118 (84.9)	29 (85.3)	1.00
Liver metastases, n (%)	62 (44.6)	17 (50.0)	0.70
Lung metastases, n (%)	64 (46.0)	14 (41.2)	0.70
CNS metastases, n (%)	63 (45.3)	9 (26.5)	0.05
Lines of previous therapy for metastatic disease, median (range)	3 (1-16)	1 (1-7)	<0.01

BMI, body mass index; ECOG, Eastern Cooperative Oncology Group performance status; HR, hormone receptor; IHC, immunohistochemistry; FISH, Fluorescence in situ hybridization; CNS, central nervous system; TKI, tyrosine kinase inhibitor; HER2, human epidermal growth factor receptor 2.
